# Improved Delay-Dependent Stability Conditions for MIMO Networked Control Systems with Nonlinear Perturbations

**DOI:** 10.1155/2014/196927

**Published:** 2014-03-10

**Authors:** Jiuwen Cao

**Affiliations:** Institute of Information and Control, Hangzhou Dianzi University, Zhejiang 310018, China

## Abstract

This paper provides improved time delay-dependent stability criteria for multi-input and multi-output (MIMO) network control systems (NCSs) with nonlinear perturbations. Without the stability assumption on the neutral operator after the descriptor approach, the new proposed stability theory is less conservative than the existing stability condition. Theoretical proof is given in this paper to demonstrate the effectiveness of the proposed stability condition.

## 1. Introduction

Complex control systems have been recently employed to control the communications among computers, controllers, and sensors due to the enlarging scale of control systems in nowadays applications [1, 2]. The real-time systems with feedback in the control loops are usually called the networked control systems (NCSs) [3]. The network time delays, induced by the transmission rate and the communication protocol of the network, are usually the source of instability of the NCSs. Therefore, the stable conditions of NCSs play an important role in the real applications.

An amount of research work has been reported in the stability analysis of the NCSs; see [4–17] and references therein. In general, the stability condition of the control systems can be categorized into two cases: the delay-dependent stability conditions [9, 10] and the delay-independent stability conditions [11–17]. For delay-dependent stability criteria, people always seek to find a delay bound which is defined as the maximum allowable delay bound that can be obtained to guarantee the NCSs stability. Based on the descriptor approach, an improved delay-dependent stability condition has been derived for a class of real multi-input and multi-output (MIMO) closed-loop networked control systems in [17]. In terms of using the linear matrix inequalities (LMI) and transferring the system to a neutral operator using the decomposition method, the maximum upper bound of the allowable delay obtained by solving a convex optimization problem by the proposed condition in [17] is larger than the results given in [11–14, 16]. However, the drawback in [17] is that the delay-dependent stable criteria are built on the stability assumption of the neutral operator. In general, we know that the stable assumption of the neutral operator limits the maximum allowable time delays, which can further affect the conservativeness of the condition. With this objective, we develop a new condition for the MIMO network control systems with multiple state time delays and nonlinear perturbations in this paper. In our new result, the stable assumption of the neutral operator is not required. We have theoretically shown that the proposed condition is less conservative than the result given in [17].

## 2. System Model and Review

Composing of a plant *G*_*p*_, a controller *G*_*c*_, and a common network with nonlinear perturbations, independent sensors, and actuators, the real multi-input and multi-output (MIMO) closed-loop networked control systems (NCSs) with multiple state time delays and nonlinear perturbations can be modeled as follows:

(1a) $$\begin{array}{c} \dot{x}\left(t\right)=Ax\left(t\right)+\overset{K}{\underset{j=1}{\sum }}A_{j}x\left(t-\tau_{j}\right)+Nf\left(x\left(t\right)\right),\;t>0, \end{array}$$

(1b) $$\begin{array}{c} x\left(t\right)=\varphi \left(t\right),\;t\in \left[-\bar{\tau },0\right], \end{array}$$

where *x*(*t*) ∈ *R* ^*n*^ is the system state vector and *A*,  *A*_*j*_  (*j* = 1,2,…, *K*) are real coefficient matrices. *φ*(*t*) is a vector-valued initial continuous function with $-\bar{\tau }\leq t\leq 0$ and the external nonlinear perturbations are assumed to be bounded in magnitude as ||*f*(*x*(*t*))|| ≤ *α*||*x*(*t*)|| for all *t* > 0. For the detailed information on the formulation of system model (1a) and (1b), one could refer to [17] and references therein.

The stability of MIMO closed-loop NCSs (1a) and (1b) has been considered in [17]. Combining with the Newton-Leibniz formula and the descriptor method by decomposing the constant coefficient matrix *A*_*j*_ of delay vector into two parts *A*_*j*_ = *A*_*j*1_ + *A*_*j*2_  (*j* = 1,2,…, *K*), system (1a) and (1b) is represented in the following equivalent expression as in [17]:

(2) y(t)=−∑j=1KAj1∫t−τjty(s)ds+(A+∑j=1KAj1)x(t)+∑j=1KAj2x(t−τj)+Nf(x(t))

with $y(t)=\dot{x}(t)$. Utilizing the coefficient matrix decomposing method, slack matrices, and linear matrix inequality (LMI), the stability criteria given in [17] have been shown more effective than several related stability theories; see references therein. However, one drawback existed in [17] is the requirement on the stability of the neutral operator; that is, the following assumption should be satisfied:

(3) $$\begin{array}{c} \overset{K}{\underset{j=1}{\sum }}\tau_{j}||A_{j1}||<1. \end{array}$$

For time delay-dependent stability criteria, it is apparent that assumption (3) imposes a constraint on the time delay, which subsequently makes the stability condition more conservative. Before presenting the new result, the stability criteria described in [17] and its corresponding brief proof are given below for convenience of presentation and comparison.

**Theorem 1 (see [17])**

Under assumption (3), the MIMO closed-loop NCS (1a) and (1b) described by descriptor system (2) is asymptotically stable for all $\tau_{j}\in \left[0,\bar{\tau }\right],\;\;(j=1,2,\ldots ,K)$ if there exist matrix *P* = *P* ^*T*^ > 0, *Q*_*j*_ = *Q*_*j*_^*T*^ > 0, *M*_*j*_ = *M*_*j*_^*T*^ > 0  (*j* = 1,2,…, *K*), *S*_1_, *S*_2_, and positive scalar *ɛ* > 0 such that

(4) $$\begin{array}{c} \Omega =\left[\begin{array}{ccccc} \omega_{11} & \omega_{12} & S_{1}^{T}{\tilde{A}}_{j2} & -S_{1}^{T}{\tilde{A}}_{j1} & S_{1}^{T}N\\ {}* & \omega_{22} & S_{2}^{T}{\tilde{A}}_{j2} & -S_{2}^{T}{\tilde{A}}_{j1} & S_{2}^{T}N\\ {}* & * & -\tilde{Q} & 0 & 0\\ {}* & * & * & -\tilde{M} & 0\\ {}* & * & * & * & -ɛI \end{array}\right]<0, \end{array}$$

where ∗ denotes the elements below the main diagonal of a symmetric block matrix and *ω*_11_ = *S*_1_^*T*^(*A* + ∑_*j*=1_^*K*^ *A*_*j*1_) + (*A*+∑_*j*=1_^*K*^ *A*_*j*1_)^*T*^ *S*_1_ + ∑_*j*=1_^*K*^ *Q*_*j*_ + *ɛα* ^2^ *I*, *ω*_12_ = *P* − *S*_1_^*T*^ + (*A*+∑_*j*=1_^*K*^ *A*_*j*1_)^*T*^ *S*_2_,$\omega_{22}=-S_{2}^{T}-S_{2}+\sum_{j=1}^{K}{\bar{\tau }}^{2}M_{j}$, ${\tilde{A}}_{j1}=[A_{11}\;\cdots \;A_{K1}]$,  ${\tilde{A}}_{j2}=[A_{12}\;\cdots \;A_{K2}]$, $\tilde{Q}=\mathrm{diag}(-Q_{1}\;\cdots \;-Q_{K})$, $\tilde{M}=\mathrm{diag}(-M_{1}\;\cdots \;-M_{K})$.

Following Lyapunov-Krasovskii functions that have been constructed in [17],

(5) V(t)=xT(t)Px(t)+∑j=1K∫t−τjtxT(s)Qjx(s)ds+∑j=1Kτj∫t−τjt∫ξtyT(s)Mjy(s)ds dξ.

In such case, the time derivative of *V*(*t*) is $\dot{V}(t)\leq \chi^{T}(t)\Omega_{1}\chi (t)$ (expression of *Ω*_1_ could be found in [17]) and *Ω*_1_ < 0 is implied by the existence of positive scalar *δ* > 0 such that (4) holds. Here, *χ*(*t*) = [*x* ^*T*^(*t*) *y* ^*T*^(*t*) *ς*_1_^*T*^(*t*) *ς*_2_^*T*^(*t*) *f* ^*T*^(*x*(*t*))]^*T*^ with *ς*_1_(*t*) = [*x* ^*T*^(*t* − *τ*_1_) ⋯ *x* ^*T*^(*t* − *τ*_*K*_)]^*T*^ and *ς*_1_(*t*) = [(∫_*t*−*τ*_1__^*t*^ *y*(*s*)*ds*)^*T*^ ⋯ (∫_*t*−*τ*_*K*__^*t*^ *y*(*s*)*ds*)^*T*^]^*T*^.

## 3. Improved Stability Condition

The above discussion shows that finding a less conservative condition which no longer relies on constraint (3) is urgent. As indicated in [17], the stability of the MIMO closed-loop NCSs is based on the stability of the neutral operator. In this section, we develop an improvement on the stability criteria for the MIMO closed-loop NCS (1a) and (1b). Before stating the result, the following lemma is needed.

**Lemma 2 (see [18])**

For a symmetric positive definite matrix *Q* ∈ *R* ^*n*×*n*^ and any matrix *A* ∈ *R* ^*n*×*n*^, the following inequality satisfies

(6) $$\begin{array}{c} 2\langle Ay,x\rangle -\langle Qy,y\rangle \leq \langle AQ^{-1}A^{T}x,x\rangle \end{array}$$

for ∀*x*, *y* ∈ *R* ^*n*^.

For the MIMO closed-loop NCS described in (1a) and (1b), we have the following theorem to ensure the stability of its solution.

**Theorem 3**

The MIMO closed-loop NCS (1a) and (1b) described by descriptor system (2) is asymptotically stable for all $\tau_{j}\in \left[0,\bar{\tau }\right],\;\;(j=1,2,\ldots ,K)$ if there exist matrix *P* = *P* ^*T*^ > 0, *Q*_*j*_ = *Q*_*j*_^*T*^ > 0, *M*_*j*_ = *M*_*j*_^*T*^ > 0  (*j* = 1,2,…, *K*), *S*_1_, *S*_2_, and positive scalar *ɛ* > 0 such that (4) holds.

**Proof**

Denote Γ(*t*) = *x*(*t*) + ∑_*j*=1_^*K*^ *A*_*j*1_∫_*t*−*τ*_*j*__^*t*^ *x*(*s*)*ds*; (4) is equivalent to the following equation:

(7) $$\begin{array}{c} \tilde{\Omega }=\left[\begin{array}{cccccc} \omega_{11} & \omega_{12} & -\omega_{11}{\tilde{A}}_{j1} & S_{1}^{T}{\tilde{A}}_{j2} & -S_{1}^{T}{\tilde{A}}_{j1} & S_{1}^{T}N\\ {}* & \omega_{22} & 0 & 0 & 0 & 0\\ {}* & * & \tilde{A} & S_{2}^{T}{\tilde{A}}_{j2} & -S_{2}^{T}{\tilde{A}}_{j1} & S_{2}^{T}N\\ {}* & * & * & -\tilde{Q} & 0 & 0\\ {}* & * & * & * & -\tilde{M} & 0\\ {}* & * & * & * & * & -ɛI \end{array}\right]<0 \end{array}$$

with $\tilde{A}=\mathrm{diag}({\tilde{A}}_{11}^{T}\omega_{11}{\tilde{A}}_{11}\;\cdots \;{\tilde{A}}_{K1}^{T}\omega_{11}{\tilde{A}}_{K1})$, $\tilde{\chi }\left(t\right)={\left[\Gamma^{T}(t)\;y^{T}(t)\;\eta^{T}(t)\;\varsigma_{1}^{T}(t)\;\varsigma_{2}^{T}(t)\;f^{T}(x(t))\right]}^{T}$ and with *η*(*t*) = [∫_*t*−*τ*_1__^*t*^ *x* ^*T*^(*s*)*ds* ⋯ ∫_*t*−*τ*_*K*__^*t*^ *x* ^*T*^(*s*)*ds*]^*T*^, which further implies there exists a positive *λ* such that

(8) V˙(t)<−λ(||Γ(t)||2+||y(t)||2+∑j=1K||x(t−τj)||2+∑j=1K||∫t−τjtx(s)ds||2+∑j=1K||∫t−τjty(s)ds||2+α2||x(t)||2).

From the definition of Γ(*t*), it is easy to know that ||*x*(*t*)|| ≤ ||Γ(*t*)|| + ∑_*j*=1_^*K*^||*A*_*j*1_∫_*t*−*τ*_*j*__^*t*^ *x*(*s*)*ds*||. With the Bunyakovskii inequality [19], this implies

(9) ||x(t)||2≤(K+1)×(||Γ(t)||2+∑j=1K||Aj1||2·∑j=1K||∫t−τjtx(s)ds||2).

Therefore, we have

(10) −||Γ(t)||2≤−1K+1||x(t)||2+∑j=1K||∫t−τjtx(s)ds||2,if  ∑j=1K||Aj1||2<1,−||Γ(t)||2≤−1(K+1)∑j=1K||Aj1||2||x(t)||2+∑j=1K||∫t−τjtx(s)ds||2, if  ∑j=1K||Aj1||2>1.

For ∑_*j*=1_^*K*^||*A*_*j*1_||^2^ < 1 and ∑_*j*=1_^*K*^||*A*_*j*1_||^2^ > 1, respectively, we choose *δ* = *λ*/((*K* + 1)[2||*A*|| + ∑_*j*=1_^*K*^||*A*_*j*_||^2^ + *Nα* ^2^ + 1]) and *δ* = *λ*/((*K* + 1)[2||*A*|| + ∑_*j*=1_^*K*^||*A*_*j*_||^2^ + *Nα* ^2^ + 1]∑_*j*=1_^*K*^||*A*_*j*1_||^2^) correspondingly. Then, we construct the Lyapunov-Krasovskii function $\tilde{V}(t)=V(t)+\delta x^{T}(t)x(t)$, where *V*(*t*) is given in (5). For ∑_*j*=1_^*K*^||*A*_*j*1_||^2^ < 1, utilizing (10) and Lemma 2, the derivative of $\tilde{V}(t)$ satisfies

(11) V~˙(t) =V˙(t)+2δ[Ax(t)+∑j=1KAjx(t−τj)+Nf(x(t))]Tx(t) ≤V˙(t)+δ[2||A||+∑j=1K||Aj||2+Nα2+1]||x(t)||2  +δ∑j=1K||x(t−τj)||2 ≤−λ(||Γ(t)||2+||y(t)||2+∑j=1K||x(t−τj)||2+∑j=1K||∫t−τjtx(s)ds||2+∑j=1K||∫t−τjty(s)ds||2+α2||x(t)||2)  +λK+1||x(t)||2+δ∑j=1K||x(t−τj)||2 ≤−λ(||y(t)||2+(1−δλ)∑j=1K||x(t−τj)||2+∑j=1K||∫t−τjty(s)ds||2+α2||x(t)||2).

Since *δ* < *λ*, it is easy to know 1 − (*δ*/*λ*) > 0 and hence $\dot{\tilde{V}}(t)<0$.

For ∑_*j*=1_^*K*^||*A*_*j*1_||^2^ > 1, the above equation can be similarly obtained. This finishes our proof.

**Remark 4**

It is apparent that the new proposed condition in Theorem 3 is not dependent on assumption (3) imposed in [17] which is used to ensure the stability of the neutral operator. Hence, Theorem 3 is less conservative than the condition given in [17].

## 4. Conclusion

An improved stability condition for multi-input and multi-output (MIMO) closed-loop networked control systems (NCSs) with multiple state time delays and nonlinear perturbations has been proposed in this paper. Without the assumption constraint to guarantee the neutral operator stability as used in [17], the new developed criteria are less conservative than the one given in [17].
